# A landscape of genomic alterations at the root of a near-untreatable tuberculosis epidemic

**DOI:** 10.1186/s12916-019-1487-2

**Published:** 2020-02-21

**Authors:** Marisa Klopper, Tim Hermanus Heupink, Grant Hill-Cawthorne, Elizabeth Maria Streicher, Anzaan Dippenaar, Margaretha de Vos, Abdallah Musa Abdallah, Jason Limberis, Matthias Merker, Scott Burns, Stefan Niemann, Keertan Dheda, James Posey, Arnab Pain, Robin Mark Warren

**Affiliations:** 1 South African Medical Research Council Centre for Tuberculosis Research, DST NRF Centre of Excellence for Biomedical Tuberculosis research, Division of Molecular Biology and Human Genetics, Faculty of Medicine and Health Sciences, Stellenbosch University, Cape Town, South Africa; 2 Global Health Institute, Epidemiology and Social Medicine, University of Antwerp, Antwerp, Belgium; 3 Sydney School of Public Health, Faculty of Medicine and Health, University of Sydney, Sydney, New South Wales Australia; 4 Pathogen Genomics Laboratory, BESE Division, King Abdullah University of Science and Technology (KAUST), Thuwal, Saudi Arabia; 5 Department of Basic Medical Sciences, College of Medicine, QU Health, Qatar University, Doha, Qatar; 6 Centre for Lung Infection and Immunity, Division of Pulmonology, Department of Medicine and UCT Lung Institute & South African MRC/UCT Centre for the Study of Antimicrobial Resistance, University of Cape Town, Cape Town, South Africa; 7 Molecular and Experimental Mycobacteriology, Research Center Borstel, Borstel, Germany; 8 Division of Tuberculosis Elimination, National Center for HIV/AIDS, Viral Hepatitis, STD, and TB Prevention, Centers for Disease Control and Prevention, Atlanta, GA 30329 USA; 9 German Center for Infection Research, Partner Site Hamburg-Lübeck-Borstel-Riems, Borstel, Germany; 10 Faculty of Infectious and Tropical Diseases, Department of Infection Biology, London School of Hygiene and Tropical Medicine, London, UK; 11 Center for Zoonosis Control, Global Institution for Collaborative Research and Education (GI-CoRE), Hokkaido University, Sapporo, Japan

**Keywords:** Tuberculosis, Drug-resistant, Beyond-XDR-TB, Missed resistance, Weakened regimen, Whole genome sequencing, Atypical Beijing, Bedaquiline

## Abstract

**Background:**

Atypical Beijing genotype *Mycobacterium tuberculosis* strains are widespread in South Africa and have acquired resistance to up to 13 drugs on multiple occasions. It is puzzling that these strains have retained fitness and transmissibility despite the potential fitness cost associated with drug resistance mutations.

**Methods:**

We conducted Illumina sequencing of 211 Beijing genotype *M. tuberculosis* isolates to facilitate the detection of genomic features that may promote acquisition of drug resistance and restore fitness in highly resistant atypical Beijing forms. Phylogenetic and comparative genomic analysis was done to determine changes that are unique to the resistant strains that also transmit well. Minimum inhibitory concentration (MIC) determination for streptomycin and bedaquiline was done for a limited number of isolates to demonstrate a difference in MIC between isolates with and without certain variants.

**Results:**

Phylogenetic analysis confirmed that two clades of atypical Beijing strains have independently developed resistance to virtually all the potent drugs included in standard (pre-bedaquiline) drug-resistant TB treatment regimens. We show that undetected drug resistance in a progenitor strain was likely instrumental in this resistance acquisition. In this cohort, ethionamide (*ethA* A381P) resistance would be missed in first-line drug-susceptible isolates, and streptomycin (*gidB* L79S) resistance may be missed due to an MIC close to the critical concentration. Subsequent inadequate treatment historically led to amplification of resistance and facilitated spread of the strains. Bedaquiline resistance was found in a small number of isolates, despite lack of exposure to the drug. The highly resistant clades also carry *inhA* promoter mutations, which arose after *ethA* and *katG* mutations. In these isolates, *inhA* promoter mutations do not alter drug resistance, suggesting a possible alternative role.

**Conclusion:**

The presence of the *ethA* mutation in otherwise susceptible isolates from ethionamide-naïve patients demonstrates that known exposure is not an adequate indicator of drug susceptibility. Similarly, it is demonstrated that bedaquiline resistance can occur without exposure to the drug. Inappropriate treatment regimens, due to missed resistance, leads to amplification of resistance, and transmission. We put these results into the context of current WHO treatment regimens, underscoring the risks of treatment without knowledge of the full drug resistance profile.

## Background

Drug-resistant tuberculosis (DR-TB) represents a global health crisis, exacerbated by TB that is resistant to most of the routinely used drugs [1–4]. Cases with resistance beyond the four drugs/drug classes defining extensively drug-resistant TB (XDR-TB, resistance to isoniazid, rifampicin, at least one second-line injectable and a fluoroquinolone) are the result of further acquisition of resistance [1–3], primary (transmitted) resistance [4] or a combination thereof [5]. Strains of the Beijing lineage of the *Mycobacterium tuberculosis* complex have previously been associated with an increased ability to develop multidrug resistance (MDR, resistance to at least isoniazid and rifampicin) and spread [6–8]. Examples are the documented outbreaks in Russia [9] and South Africa (Gauteng Province) [10], as well as the widespread transmission of a highly resistant strain in the Eastern Cape (EC) Province of South Africa [4]. The latter strains belong to the atypical (ancient) subgroup of Beijing strains, also termed Asia Ancestral 1 [11], ST11 [12], Lineage 2.2.2 [13], etc. [14], and are distinguished from typical (modern) Beijing strains primarily through the absence of an IS*6110* in the NTF-1 region (so designated by Plikaytis et al. [15]). This genotype is usually seen at low frequency worldwide, with the notable exception of Japan, Vietnam and Taiwan [16–20]. Similarly, drug-susceptible atypical Beijing strains are generally present at low frequency in South African settings [21]. However, in the EC, the atypical Beijing strains are over-represented among drug-resistant TB strains [4]. Furthermore, an increasing incidence of atypical Beijing strains observed in the Western Cape (WC) Province, in particular among XDR-TB patients [21], suggests an influx through migration from the EC. However, detailed studies have not yet been performed. These data suggest a potential survival advantage in drug-resistant atypical Beijing isolates from the region, which enhances their ability to transmit and cause disease, as well as overcome the potential fitness cost associated with drug resistance [22, 23].

We aimed to interrogate the genomes of highly resistant atypical Beijing strains (resistant to up to 13 drugs, Additional file 1) from the EC and WC through whole genome sequencing (WGS), which provides a thorough and unbiased understanding of genome features pertaining to the evolution of mycobacterial strains. Our analysis included a small number of presumed drug-susceptible isolates of the same genotype, as well as published [11, 24, 25] and unpublished genome sequences from typical and atypical Beijing strains isolated from other South African regions and from different settings across the globe to describe evolutionary relationships.

## Methods

### Strain selection

In order to determine whether genomic changes account for the apparent increased ability to acquire resistance and spread, clinical isolates of the atypical Beijing genotype isolated from patients residing in the EC (*n* = 60) and WC (*n* = 92), sampled between 1994 and 2016 (Additional file 2), were included in the study. Isolates originating from the EC were selected for WGS based on their genotypic (Sanger sequencing) drug resistance profiles [4], reflecting the available diversity in terms of number and type of mutations detected. Subsequently, our sequence database, containing sequences of many different studies and originating mostly from the WC, was queried for sequences of the Beijing genotype, based on Spolpred [26] results. The selection was a convenience sample, making use of available strains collected for various studies, reflecting both an approximation of the true population structure, and genomic variety. Only a small number (*n* = 7) of presumed drug-susceptible (based on routine phenotypic drug susceptibility testing (DST) and limited Sanger sequencing) atypical Beijing isolates with high-quality sequences were available, due to its low prevalence in the population. Treatment history and outcomes are unknown for all patients sampled. Additional genome sequences analysed in this study comprised of a selected variety of published Beijing strains originating from South Africa and other global settings [11, 24, 25]. The final selection (*n* = 59) was made to represent only a small number of each available typical Beijing subclade. These strains were included to determine the phylogenetic relationship of South African Beijing strains compared to global representatives of Beijing genotype strains and to determine changes that are unique to the atypical Beijing clade (Additional file 2).

### DNA sequencing

Clinical isolates were cultured under biosafety level 3 conditions on 7H10 media. The bacteria were heat-killed prior to standard phenol/chloroform DNA extraction [27]. Paired-end genomic libraries were prepared using either TruSeq DNA Sample Preparation Kits V2 (Illumina Inc., San Diego, CA, USA) or NEBNext Ultra DNA library prep kit for Illumina (New England BioLabs) per manufacturers’ recommendations. Pooled samples were sequenced on an Illumina HiSeq 2000 or NextSeq 550, respectively.

### DNA sequence analysis

The resultant paired-end sequencing data, as well as published raw reads, were analysed using an in-house sequence analysis pipeline, as described by Black et al. [28]. Briefly, Trimmomatic [29] was used to trim reads with a sliding window approach and an average phred score of 20, prior to alignment to *M. tuberculosis* H37Rv (GenBank NC000962.2) with three different algorithms, namely Burrows-Wheeler aligner, NovoAlign and SMALT [30–32]. The Genome Analysis Tool Kit (GATK) [33] and Samtools [30] were used for variant calling, while GATK was also used to identify areas of zero coverage (areas deleted from the genome). Drug resistance conferring mutations were identified using a reference library [34]. Only high-quality sequences, based on average read depth and percentage mapped reads, and variants called by all combinations of alignment software and variant callers were used in further analyses (Additional file 2). Alignments of the different strains were inspected visually with Artemis (Sanger Institute) [35] and Genomeview [36] to inspect boundaries of large deletions. Large deletions were considered to be true when there was a clear cut in stacked reads with no reads covering the deleted region in Bamview in Artemis. Apparent deletions, where some low-depth reads were present, were judged individually by comparing the region to that of other strains to gauge the reliability of sequencing of the region. Where coverage of a region seemed haphazard (e.g. in repetitive regions), they were considered to have a wild-type genotype, as were apparent deletion of genes that are noted to have high sequence similarity to other genes in the *M. tuberculosis* genome.

### Phylogeny

A sequence consisting of concatenated high-confidence sequence variants (from coding and non-coding sequence) was prepared from each isolate. Known drug resistance conferring variants as described by Coll et al. [37], variants located in repeat regions, with quality scores generated by Samtools below 200, per-base coverage of less than 10 reads or heterogeneity frequency below 0.8 were removed prior to compiling the concatenated sequence. Cutoff values were chosen to result in high-confidence variant sites, which were subsequently written to a multi-FASTA alignment, which in turn was used for phylogenetic inference in IQ-TREE v1.5 [38]; gaps were excluded. ModelFinder [39] identified K3Pu+ASC+R4 as the most likely substitution model, and the Maximum Likelihood tree was reconstructed accordingly with 1000 standard nonparametric bootstrap replicates. *M. tuberculosis* H37Rv, accession NC000962.2, was used as an outgroup [40], but is not shown on the figure. The subsequent tree was annotated with drug resistance mutations, using the *ggtree* package in R [41]. Clades were assigned based on the topology of the tree, but also taking drug resistance markers into account.

We performed linear regression analysis on the whole tree, as well as on the AA1SA clade only, to determine if a correlation exists between branch length and average coverage. Additionally, we did a Student’s *t* test to determine whether read length (100 bp on Illumina HiSeq 2000 or 150 bp on Illumina NextSeq 550) influenced average branch length.

It should be noted that within the context of this study, we use the term “transmission” not in the sense of direct person-to-person transmission, but rather reflecting past and more recent events within an endemic setting.

### Comparative genomics

**Fig. 1:**
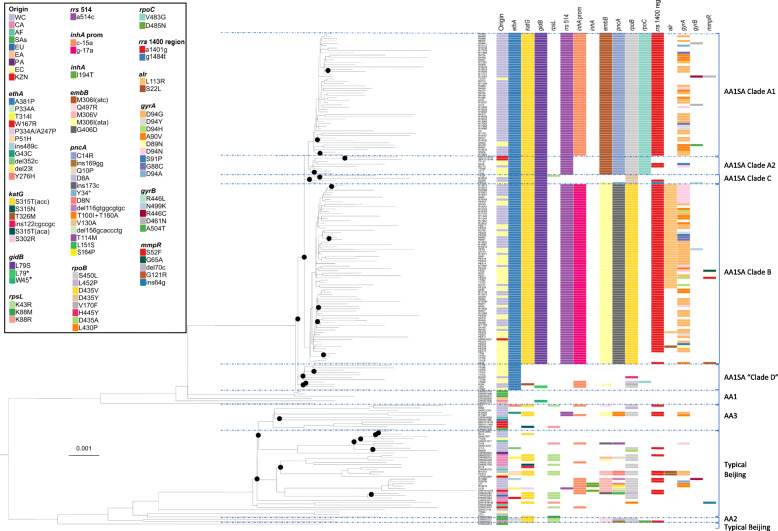
The annotated Maximum Likelihood phylogeny of various Beijing-family *M. tuberculosis* strains to demonstrate the relative position and drug resistance mutation profiles of South African isolates (AA1SA) belonging to the Asian Ancestral 1 clade. The phylogeny indicates that the branching of AA1 is the most ancient in the Beijing lineage, and suggests that various forms of Beijing was introduced into South Africa independently. It appears that only one introduction of AA1 occurred, which subsequently evolved into different subclades. Clades: AA1SA, Asian Ancestral 1 South Africa; AA1, Asian Ancestral 1; AA2, Asian Ancestral 2; AA3, Asian Ancestral 3. Asian Ancestral clades collectively comprise atypical Beijing, while the remainder of the clades represent various forms of typical Beijing. Geographic origins: EC, Eastern Cape; WC, Western Cape; KZN, KwaZulu-Natal; CA, Central Asia; EA, Eastern Asia; SAs, Southern Asia; EU, Europe; PA, Pacific; AF, Africa. Drug resistance mutations are organised according to gene and type of resistance caused: *ethA*, ethionamide; *katG* and *inhA*, isoniazid; *gidB*, *rpsL* and *rrs* 514-region, streptomycin; *inhA* prom(oter), isoniazid and ethionamide; *embB*, ethambutol; *pncA*, pyrazinamide; *rpoB*, rifampicin; *rrs* 1401-region, amikacin, kanamycin, capreomycin; *alr*, terizidone/cycloserine; *gyrA* and *gyrB*, fluoroquinolones; *mmpR*, bedaquiline and clofazimine. We show all observed *mmpR* mutations, as the role of these in conferring resistance is not well documented, although several different mutations in *mmpR* has been implicated in resistance. Nodes with a bootstrap support of 70 or more are indicated by black circles. The phylogeny is rooted to H37Rv

A SNP distance matrix was produced by comparing the variants found between strains. This included variants used in the phylogenetic analysis as well as drug resistance causing mutations. A similar approach was used to identify variants that occurred uniquely in different phylogenetically assigned groups, but this analysis included small insertions and deletions. Thus, the phylogeny, which did not include drug resistance causing mutations or insertions and deletions, was used to inform grouping for further analysis which did include these variants. Briefly, an in-house Python script was used to calculate the number of variants unique to a selected group of isolates (e.g. Clade A in Fig. 1), compared to another group of isolates (e.g. Clade B in Fig. 1). The output consists of three lists: (a) variants unique to the group of interest, (b) variants unique to the comparator group and (c) variants present in both groups. The first and second lists (variants unique to each group) were inspected for variants that are present in all members of a given group, and the sum of these was taken to be the minimum inter-clade distance. Additionally, in the above example, variants that occurred in all clade A and B isolates represent ancestral variants, while variants that occurred in both groups, but not in all members of either group, were considered homoplastic. Variants occurring in all isolates from a specific group, and not in other investigated isolates, were considered defining of the group in question.

In a separate analysis, we inspected sequences for known resistance-causing mutations that occurred at frequencies lower than our 0.8 cutoff for the phylogeny and comparative genomics, to detect emerging resistance.

### Variant analysis

Protein Variation Effect Analyzer (PROVEAN) v1.1 [42] was used to predict whether individual variants that were defining of a specific phylogenetic group would disrupt protein function.

### Minimum inhibitory concentration determination for ethionamide, streptomycin and bedaquiline

A selection of isolates with an *ethA* A381P mutation was used to determine the minimum inhibitory concentration (MIC) of ethionamide (ETH) in the presence or absence of *inhA* promoter mutations. MIC testing was done at 5, 20 and 40 μg/ml ETH in a MGIT 960 BACTEC™ (BD Diagnostic Systems, NJ, USA) instrument and results analysed with Epicentre™ software. *M. tuberculosis* H37Rv (ATCC 27294) was used as a fully susceptible control.

Similarly, additional isolates were selected based on the presence of mutations associated with streptomycin (SM) resistance, to determine the effect of *gidB* L79S mutations at 0.5, 1 and 2 μg/ml SM on MIC.

Lastly, one isolate with a mutation in *mmpL5* was available for bedaquiline (BDQ) resistance testing at the following concentrations: 0.125, 0.25, 0.5, 0.75 and 1 μg/ml. Drug dilutions were prepared in polystyrene tubes.

## Results

### Phylogeny

A maximum likelihood (ML) phylogeny was generated to contextualise South African Beijing strains in the global perspective, focusing on the atypical Beijing group called Asia Ancestral 1 (AA1), by Merker et al. [11] (Fig. 1). The phylogenetic tree generated was based on 4627 variable sites (selection described in methods) in 211 isolates and was considered robust, with bootstrap values well above 70 at all major branches, and in broad agreement with published phylogenies [11]. The phylogeny showed that South African Beijing strains (including typical and atypical) are interspersed with strains from other global settings. Furthermore, some individual branches contain strains from different global locations. These results suggest multiple introduction events of Beijing strains into South Africa.

The South African strains of the AA1 genotype (Fig. 1) have distinct features (described below) compared to those identified elsewhere and broadly correspond to Beijing sublineage 1 as described by Hanekom et al. [7]. For the purpose of this study, we propose to call this clade AA1SA. Our phylogenetic analysis indicates that this monophyletic Beijing clade consists of (sub)clades A through D collectively (Fig. 1) and its close relation to the AA1 strains was confirmed by the presence of all of AA1-definitive SNPs reported by Merker et al. [11]. Our phylogeny further affirms that the branching point basal to Asian Ancestral 1 (AA1) is the most ancient within the Beijing strain family (Fig. 1). While AA1SA are abundant in the EC and WC, a limited number was recorded by Cohen et al. [25] in KZN, as expected based on the strain type distribution of the respective provinces [21, 25]. Our analysis also revealed that within subclades of AA1SA, pairwise SNP distance is variable. In some instances, it is relatively low, considering the wide temporal and geographical space of sampling: 88 isolate pairs had a SNP distance of < 30. In the remaining isolates, the SNP distance ranged from 31 to 286. A SNP distance matrix is presented in Additional file 3. This variability is also evident in the terminal branch lengths of the phylogeny. We performed statistical analyses to determine whether the variability in branch length may be an artefact related to the average coverage or read length. Linear regression analysis for average coverage and terminal branch length indicates an *R*^2^ of 0.016 when considering the entire tree and 0.188 when only the AA1SA genomes were included, suggesting no correlation. Similarly, there was no difference in average branch length comparing read lengths of 100 bp vs 150 bp (*P* > 0.05). Accordingly, we conclude that neither average coverage nor read length is responsible for the observed variable branch lengths.

### Variants defining the AA1SA genotype

The AA1SA sublineage described here is defined by 86 AA1SA-specific variants, which distinguish it from all other Beijing isolates investigated. This includes SNPs and small insertions or deletions (Additional file 4) as well as three large deletions (Table 1). Of the 86 SNPs, 45 (52.3%) were non-synonymous mutations (including 3 frameshift mutations) in coding regions, 26 (30.2%) were synonymous and 14 (16.2%) were intergenic. Twelve SNPs were found to be likely deleterious by PROVEAN [42] analysis. Of these, 9 were in non-essential genes with known or unknown function (Table 2), namely *Rv1877*, *ethA*, *desA3*, *cut5B* and *Rv2303c* (known function), and *Rv0421c*, *Rv1053c*, *Rv1907c*, and *Rv2923c* (unknown function). Essential genes harbouring predicted deleterious SNPs were *mprB* (a two-component sensor kinase), *ompA* (an outer membrane protein) and *ruvA* (a Holliday junction DNA helicase)*.*

**Table 1:** Genomic locations of observed large deletions in AA1SA isolates

Coordinates	Size (BP)	Genes affected	Corresponding RD	RD coordinates	Presence	Notes
2128380–2129581	1202	*glnA3*, *Rv1879* (glutamine synthesis)	163	2127981–2128972	AA1SA, AA1	Additional mutations were found in other genes involved in glutamine synthesis (*glnE* ACG278ACA and *glnA2* CTG117TTG) in all AA1SA strains
2090364–2090443	81	*Rv1841c*	–	–	AA1SA	Non-essential conserved hypothetical membrane protein
2263779–2266164	2385	*Rv2016*–*Rv2019*	175a	2263448–2263637	AA1SA, some AA1	*Rv2016*, *Rv2018* and *Rv2019* are non-essential, conserved hypothetical proteins. *Rv2017* is a transcriptional regulator, and essential for in vitro growth [43].

*BP* base pairs, *RD* Region of Difference; from Tsolaki et al. [44]

**Table 2:** Deleterious mutations found in all AA1SA isolates

Amino acid change	Gene	Product	Function	PROVEAN score*	Essentiality
P251L	*ompA* (*Rv0899*)	Outer membrane protein A	Porin of low specific activity	− 7.220	Essential
G59D	*Rv1877*	Conserved integral membrane protein	Involved in transport of drug across the membrane	− 6.971	Non-essential
D53G	*cut5b* (*Rv3724B*)	Probable cutinase Cut5b	Hydrolysis of cutin (a polyester that forms the structure of plant cuticle)	− 5.649	No info
Q103R	*Rv0421c*	Conserved hypothetical protein	Unknown	− 2.857	Non-essential
R27H	*Rv1053c*	Hypothetical protein	Unknown	− 5.000	Non-essential
P141S	*Rv1907c*	Hypothetical protein	Unknown	− 3.922	Non-essential
A46V	*Rv2923c*	Conserved protein	Unknown	− 3.437	Non-essential
R39W	*ruvA* (*Rv2593c*)	Probable Holliday junction DNA helicase	Mediates Holliday junction migration by localised denaturation/reannealing	− 6.219	Essential
A381P	*ethA* (*Rv3854c*)	Monooxygenase	Activates the pro-drug ethionamide	− 4.576	Non-essential
D304N	*mprB* (*Rv0982*)	Two-component sensor kinase	Sensor part of a two component regulatory system (MPRAB system)	− 4.284	Essential

*Mutations with scores below − 2.5 were considered deleterious

The large deletions observed in all AA1SA isolates (Table 1) include an 81-bp deletion in *Rv1841c*, a 1202-bp deletion from the region *glnA3*-*Rv1879* and a 2385-bp deletion from *Rv2016*-*Rv2019*. The latter two deletions encompass Region of Difference (RD) 163 and RD175a [45], respectively. However, the boundaries of the deletions observed here and the previously described RDs are very different, suggesting that these were separate events. None of these deletions was found in any investigated Beijing strains outside of the AA1 genotype.

### AA1SA subclades

**Fig. 2:**
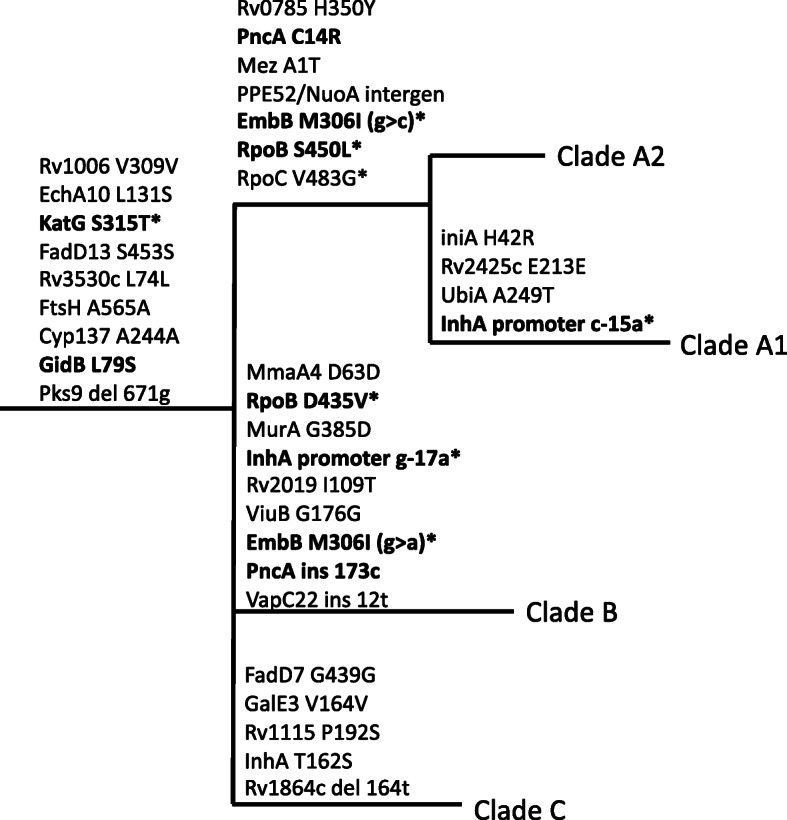
Schematic representation of variants defining the AA1SA subclades A–C, not drawn to scale. Variants indicated in boldface are associated with drug resistance. Variants labelled with an asterisk (*) also occur outside of these branches, but not necessarily elsewhere in the phylogeny shown in Fig. 1

It appears that a single AA1SA progenitor was introduced into South Africa. After introduction into South Africa, the AA1SA genotype diversified into four subclades (clades A, B, C and D, with clade A further subdivided into A1 and A2 (Fig. 1)). While clade D is not monophyletic within AA1SA, we treat it as such for the purpose of comparison, as its members have at least two things in common, which is not shared by clades A to C, namely the apparent lack of transmissibility and the limited number of drug resistance mutations acquired. Clades A, B and C appear to have simultaneously diverged from the same common progenitor, as supported by SNP data. However, the near-zero internal branch lengths at the base of these clades should be interpreted with caution; ML could not resolve this apparent polytomy. While the three clades display sequence commonality, each clade has distinct defining variants (Fig. 2, Additional file 5). Subclades A2 and A1 are sister taxa, as indicated by the phylogeny (Fig. 1), and supported by the defining variants of each subclade (Fig. 2, Additional file 5). While clades A1 and A2 have seven variants in common that differentiate them from clades B and C, clade A1 has four additional variants that in turn differentiate it from clade A2. Although Clade D is in fact polyphyletic, for the purposes of discussion, it is regarded as a single sister taxon to clades A, B and C collectively.

Each of the AA1SA subclades evolved a unique drug resistance mutation profile, including two major subclades (clades A and B, Fig. 1) of highly drug-resistant strains exhibiting strong clonal characteristics. These clades have evolved from a common progenitor with a minimum inter-clade distance of 17 SNPs. Clades A and B each has a unique subset of known drug resistance (DR) mutations (Fig. 1), and although these DR mutations were excluded from the phylogenetic analysis, clustering of strains into subclades was concordant with DR mutation profile. Clade A2 is a sister taxon of A1 and accordingly shows a subset of A1’s drug resistance markers (Fig. 1), lacking the *inhA* promoter -15 mutation in all cases, as well as the *rrs* 1401 mutation in the majority.

All clade C and D isolates had a number of drug resistance mutations, in addition to the AA1SA-defining *ethA* A381P mutation (Fig. 1). The *katG* S315 T mutation occurred in all clade C, but not clade D isolates, although this mutation is known to be highly homoplastic and is frequently observed in various strain types. Further resistance mutations do not appear to conform to a clear pattern within the phylogeny, suggestive of limited transmission.

Special attention was drawn to the sequence of emergence of further drug resistance mutations leading to beyond-XDR phenotypes. Although “beyond-XDR” is not an officially recognised term, we use it to broadly describe strains that are resistant to additional first-, second- and third-line drugs not included in the simplest definition of XDR, emphasising the compounded nature of resistance present. The phylogenomic inference (Fig. 1) suggests that the most deeply rooted drug resistance mutation within AA1SA was *ethA* A381P, followed by *katG* S315T and *rrs* 514 a>c mutations causing ETH, isoniazid (INH) and SM resistance, respectively. Interestingly, a previously undescribed non-synonymous *gidB* L79S mutation likely emerged in the progenitor of clades A, B and C, around the same time of the first occurrence of the *katG* mutation and before the *rrs* 514 mutation. Subsequently, different clade-specific mutations in *rpoB*, *embB*, *pncA* and *inhA* promoter were acquired, conferring resistance to rifampicin (RIF), ethambutol, pyrazinamide and INH and ETH, respectively. Within clade B, the chronology of acquisition of these four mutations is indiscernible. However, in clade A1, the *inhA* promoter mutation appears to have occurred last of these four mutations, based on the absence of the *inhA* promoter mutation in clade A2 strains. In a subset of clade B isolates, an *alr* L113R mutation, conferring D-cycloserine (CYC) and terizidone (TZD) resistance [46], occurred after the afore-mentioned mutations (Fig. 1). *rrs* 1401 a>g mutations seen in clades A, B and C likely occurred before the observed variety of *gyrA* mutations, suggesting clonal expansion at pre-XDR level.

Our stringent filtering settings excluded any variant occurring at a read frequency less than 0.8 at the given genomic position for each isolate. Analysis of variants occurring at lower frequency (< 0.8) revealed that this method misses approximately 5% of fluoroquinolone resistance within the sample set, as well as a small number of other resistances (Additional file 6).

### Minimum inhibitory concentration (MIC) determination for ethionamide, streptomycin and bedaquiline

ETH MIC testing in BACTEC MGIT 960 confirmed that all (*n* = 15) tested isolates carrying the AA1SA-specific *ethA* A381P mutation have ETH MICs above the critical concentration (5 μg/ml) [47] despite the lack of *inhA* promoter mutations in several (12/15) of these isolates (Table 3). The presence of an *inhA* promoter mutation in addition to an *ethA* mutation did not appear to increase the MIC at the concentrations tested.

**Table 3:** MIC of isolates with the *ethA* A381P mutation

Isolate	MGIT result (cc = 5 μg/ml)	In phylogeny	*inhA* promoter
TT372	5–20 μg/ml	Yes	WT
TT679	5–20 μg/ml	Yes	WT
R3239	5–20 μg/ml	Yes	WT
TT545	> 20 μg/ml	Yes	WT
TT574	> 20 μg/ml	Yes	WT
TT607	> 20 μg/ml	Yes	WT
TT606	> 20 μg/ml	No	WT
TT589	> 20 μg/ml	Yes	WT
R4863	> 20 μg/ml	Yes	WT
SAWC6519	> 20 μg/ml	Yes	-15
R11121	> 20 μg/ml	Yes	-15
R13931	> 20 μg/ml	Yes	-17
R6768	> 20 μg/ml	No	WT
R9402	> 20 μg/ml	No	WT
R10010	> 20 μg/ml	No	WT
H37Rv (no mutation control)	< 5 μg/ml	Yes	WT

MIC testing for SM resistance demonstrated MICs of < 1 μg/ml for isolates with wild-type *gidB* and no other SM resistance associated mutations (*n* = 6); 1 μg/ml for isolates with the *gidB* L79S mutation, but lacking other known SM resistance causing mutations (*n* = 2), and ≧ 2 μg/ml for isolates with both the *gidB* mutation and an additional known SM resistance causing mutation (*n* = 4) (Table 4). The critical concentration (CC) for SM in MGIT 960 is 1 μg/ml [47, 48]; thus, all tested isolates with the *gidB* mutation were resistant to SM. However, an MIC close or equal to the CC is likely to be missed during routine susceptibility testing due to inter-experiment variability. Therefore, for the purpose of this work, we regard an MIC of 1 μg/ml as “low-level” resistance, compared to “high-level” resistance of at least double the CC.

**Table 4:** Minimum inhibitory concentrations of streptomycin for strains with a *gidB* L79S mutation

Isolate	MGIT result (cc = 1 μg/ml)	Mutations
H37RV (control)	0.5 μg/ml	–
H37MA (control)	≤ 0.5 μg/ml	–
R296	≤ 0.5 μg/ml	No *gidB*
R3239	≤ 0.5 μg/ml	No *gidB*
TT372	≤ 0.5 μg/ml	No *gidB*
TT648	≤ 0.5 μg/ml	No *gidB*
R9248	1.0 μg/ml	*gidB* L79S
R18832	1.0 μg/ml	*gidB* L79S
TT17	> 2.0 μg/ml	*gidB* L79S + *rrs*514c
TT649	> 2.0 μg/ml	*gidB* L79S + *rrs*514c
TT321	> 2.0 μg/ml	*gidB* L79S + *rpsL* K43R
PES16	2.0 μg/ml	*gidB* L79S + *rrs*1484t

The critical concentration of BDQ in MGIT was taken to be 1 μg/ml [48]. One isolate, with a G121R mutation, was shown to be resistant at 4 μg/ml. This mutation, as well as S52F, was predicted to be deleterious by PROVEAN analysis, while G65A was predicted to be neutral.

## Discussion

We report the development of beyond-XDR-TB via multiple evolutionary paths. These findings are supported by our phylogenomic analysis showing that the atypical Beijing clade named AA1SA here appears to originate from a single AA1-clade progenitor. Furthermore, the AA1SA strains are closely related, resembling an outbreak which has been spreading for more than a decade and is present in at least three South African provinces (Fig. 1). Taken together, these factors suggest that this strain is now endemic. Wide variation in terminal branch lengths is observed and is believed to be a reflection of the wide geographic and temporal sampling space. Sequencing error, which would be random, did not contribute to the variable branch lengths, given our stringent variant quality assurance, including a heterogeneity cutoff of 0.8. Furthermore, no statistical evidence could be found for read length or average coverage to influence branch lengths.

The phylogeny further shows the AA1SA clades A through D in agreement with genomic drug resistance marker combinations. This congruence supports the phylogeny, as drug resistance markers were excluded for its inference. The phylogeny also indicates that these drug resistance marker combinations evolved parsimoniously rather than on multiple independent occasions, thereby suggesting the scenario that is more likely form an evolutionary perspective.

We identified variants that are specific for AA1SA strains, including large deletions that may be useful for the identification of AA1SA strains. Interestingly, one of the deletions includes *Rv2017*, thought to encode a transcriptional regulator and to be essential for in vitro growth [43]. The finding that this gene was deleted questions the definition of essentiality by Himar-1 transposition.

Deleterious SNPs defining of AA1SA strains include variants in genes with roles in transport of drugs across the membrane (*Rv1877*) [49], macrotetrolide resistance (*Rv2303c*; based on cross-species protein similarity) [50], pathogenesis and reactivation from latent infection (two-component sensor kinase, *mprB*) [51] and the entry of hydrophilic molecules into the bacterial cell (*ompA*) [52]. Interestingly, in addition to the deleterious mutations, a synonymous SNP in the latter gene (CAG276CAA) also occurs in all the AA1SA strains (Additional file 4). We propose that these gene mutations may all be plausible candidates for contributing to a phenotype that may be better adapted to gain drug resistance mutations and survive the fitness cost thereof. However, roles of these variants need further investigation and while we comment on deleterious mutations, we do not understand potentially advantageous mutations.

AA1SA strains of clades A1 and B independently acquired drug resistance mutations beyond the definition of XDR-TB from a highly similar genomic background, suggesting an inherent ability to overcome associated fitness cost. This is further affirmed by the ability to spread, as suggested by the large number of closely related isolates in each clade. Additional variants with currently unknown roles uniquely occur in each clade (Fig. 2; Additional file 5) and may contribute to the robust phenotypes which are able to accumulate resistance and spread. Although drug resistance mutations were excluded from the phylogenetic analysis, the majority of isolates still clustered into clades A1 and B as would be expected based only on known drug resistance mutations (Fig. 1), suggesting an outbreak of drug-resistant strains. Transmission within both clusters A1 and B appears to occur at pre-XDR level, followed by independent acquisition of fluoroquinolone resistance, as is evident from the variety of *gyrA* mutations (Fig. 1). However, the *rrs* 1401 a>g mutation represents the most common mechanism of second-line injectable resistance. Therefore, acquisition of this mutation on multiple occasions cannot be ruled out. While the drug resistance mutations in clade A1 isolates, *inhA* promoter -15 c>t and *rpoB* S450 L (*E. coli* S531 L), as well as the compensatory mutation *rpoC* V483G individually are observed frequently across lineages [53], the corresponding mutations in clade B (*inhA* promoter -17 g>t and *rpoB* D435V [*E. coli* D516V]) are rare outside of this lineage.

*KatG* or *inhA* promoter mutations can occur independently as is expected from homoplastic variants (Fig. 1). However, all of clades A, B and C have the same *katG* mutation, supporting our assessment that *katG* mutations arose before *inhA* promoter mutations in these clades. Although this is the most frequently observed *katG* mutation, further support can be found in our earlier work [4], which shows the likelihood of the *katG* mutation arising before the *rrs* 514- and *inhA* promoter mutations.

A *gidB* L79S mutation that confers SM resistance close to the critical concentration is present in clades A, B and C. Certain mutations in *gidB* have been reported to lead to low-level SM resistance, while dramatically increasing the probability of acquisition of high-level SM resistance by the *rrs* 514 a>c mutation [54]. In the presence of historic treatment regimens [55], the *gidB* mutation reported here may have similarly led to the acquisition of additional mutations in *rrs* or *rpsL*, conferring higher levels of SM resistance, and thereby weakening the regimen. This may have led to step-wise acquisition of further resistance in the absence of appropriate susceptibility testing and adaptation of treatment. Within clade C, various combinations of drug resistance mutations evolved, lending credence to the notion that the *gidB* mutation may trigger resistance acquisition. However, it appears that very little transmission of these clade C genotypes occur, as supported by our previous work showing low abundance of strains with these drug resistance profiles [4]. In contrast, clades A and B were highly successful, based on the amount of transmission observed. *inhA* promoter mutations appear to contribute to this success when comparing the relative abundance between clades A1 with and A2 without an *inhA* promoter mutation. However, this observation needs to be validated by epidemiological studies.

Interestingly, *inhA* promoter mutations do not make a difference in the resistance pattern of either clade A1 or B, in the presence of both *katG* and *ethA* mutations, that arose before the *inhA* promoter mutations. Given that *inhA* promoter mutations rarely occur in the absence of any other drug resistance mutation and that they appear to be a gateway to XDR phenotypes [56], we propose that these mutations have a compensatory role in addition to causing drug resistance. This demands further investigation into the role of an *inhA* promoter mutation in a background of ETH- and high-level INH resistance. Similarly, an *inhA* gene mutation occurs in all clade C isolates (Fig. 2; Additional file 5). However, this mutation appears to be neutral according to PROVEAN analysis and has not specifically been associated with INH resistance to our knowledge. Given the co-occurrence of a *katG* mutation in the affected strains, site-directed mutagenesis would be required to determine its role in drug resistance.

We were surprised to find that the first drug resistance mutation acquired was *ethA* A381P (Fig. 1), which is associated with ETH resistance [57], a drug widely used in second-line treatment regimens. Interestingly, a similar observation was made in an MDR-TB outbreak originating in the Horn of Africa, where a capreomycin resistance conferring *tlyA* mutation was found to be present in otherwise susceptible progenitors [58]. While it is possible that the *ethA* mutation simply arose by chance, ETH was used in the past (since the 1960s) in non-standardised therapy, including first-line therapy [59, 60], which may explain the early acquisition and therefore deeply rooted evolution of this resistance marker. Thus, the fixed nature of the marker could explain ETH resistance in recent patients who should be ETH-naïve according to South African guidelines [61]. The presence of the marker in all investigated strains of this genotype indicates that the ancestral strain most likely either had the *ethA* mutation on introduction to the region or acquired it soon after.

Under South African guidelines at the time when samples used in this study were collected [61], if RIF resistance was present (either through acquisition or transmission) and identified, the patient would be treated with an ETH-containing second-line regimen without routine susceptibility testing that would detect resistance by *ethA* mutations. Under these conditions, ETH-resistant strains would acquire additional resistance more readily due to an inadvertently compromised drug regimen. This is supported by the comparatively large proportion of MDR- (27%) and pre-XDR- and XDR-TB (93%) strains of the AA1SA genotype reported in the EC [4], which can be explained by the inability of the standard MDR regimen at the time to control these strains that are already resistant to at least one second-line drug (ETH), as well as the companion drugs pyrazinamide and ethambutol. Inefficient treatment in turn leads to extended infectiousness and transmission, perpetuating the epidemic. Therefore, the contribution of the *ethA* mutation to the epidemic is likely due to suboptimal diagnostic and treatment algorithms rather than a mutation-specific physiological mechanism. While site-directed mutagenesis to prove causality remains to be done, it was confirmed by MIC determination that all tested isolates with the *ethA* mutation, and without *inhA* promoter mutations, were indeed resistant to ETH, supporting the association with resistance.

A recent study of beyond-XDR-TB patients, including patients infected with AA1SA strains, noted that 63% of beyond-XDR patients were discharged from hospital, having no further treatment options in the pre-bedaquiline era. Of these, 60% had an unfavourable outcome and 21% survived for more than 12 months, suggesting prolonged exposure of contacts [24]. In June 2018, the South African Health Ministry announced bedaquiline (BDQ) containing regimens for all RIF-resistant TB cases. While the decision was widely praised, in most cases, BDQ will be prescribed without full knowledge of available effective drugs when routine testing is done only for INH, RIF, ofloxacin (OFX) and amikacin (AMK), placing the long-term usefulness of the drug at risk. While we did not conduct comprehensive BDQ testing, literature reports variable association between BDQ resistance and a large variety of different *mmpR* mutations, and frameshift mutations in general appear to cause greater increases in MIC than amino acid changes [62]. The S52F mutation observed in our cohort was reported by Villellas et al. to be associated with BDQ resistance [63], and our own results suggest at least one more BDQ-resistant case. Therefore, we advocate caution when prescribing BDQ in patients infected with strains harbouring *mmpR* mutations. In Table 5, we present the 2018 WHO treatment guidelines and show for clades A1 and B the percentage of patients that would still benefit from each medicine. The majority of cases will not benefit from fluoroquinolones or most of the group C medicines. Based on the common mutation profile, patients infected with clade A1 strains are likely to benefit from a regimen composed of BDQ, linezolid, clofazimine and CYC/TZD, with the potential addition of delamanid (DLM). However, in a few cases, cross-resistance to BDQ and clofazimine necessitates the addition of a carbapenem or *p*-aminosalicylic acid (PAS). In contrast, less than half of the patients infected with clade B will benefit from the same regimen, due to widespread resistance to CYC/TZD. While no known genetic resistance markers for PAS was found in the cohort, up to 20% of XDR-TB patients in an Eastern Cape study were phenotypically resistant to the drug [4]. These data demonstrate that at best some beyond-XDR-TB (clade A1 or B infected) patients can still be treated with up to six effective anti-TB drugs, plus adjunctive agents. In contrast, some patients may have as little as two effective anti-TB drugs, plus adjunctive agents left for treatment, prompting consideration for how to treat these patients. A recently published trial questions the value of DLM in conjunction with an optimised background regimen [65]. Moreover, the DLM containing regimen will be further compromised during the continuation phase when BDQ and DLM is discontinued. A regimen containing fewer than four effective drugs carries the risk of losing the value of new potent drugs due to resistance acquisition, e.g. by mutations in *rv0678* as recently reported [66]. It should also be noted that the majority of isolates in our cohort were sampled prior to the availability of BDQ and DLM. Thus, while it is likely an accurate representation of pre-existing resistance, the introduction of these drugs in routine care may increase the risk of emergence of resistance to BDQ and DLM.

**Table 5:** Recommended drug regimens and predicted effectivity for XDR AA1SA strains

2018 WHO-recommended grouping of MDR-TB drugs [64]		Effectivity in AA1SA strains	
WHO grouping	Anti-tuberculous drug	Clade A1	Clade B
		% cases that would benefit	% cases that would benefit
Group A: include all three medicines where possible	Levofloxacin *OR* moxifloxacin	27%*	22%*
	Bedaquiline	98%	96%
	Linezolid	100%	100%
Group B: add one or both medicines	Clofazimine	98%	96%
	Cycloserine *OR* terizidone	100%	40%
Group C: add to complete the regimen and when medicines from Groups A and B cannot be used	Ethambutol	0%	0%
	Delamanid	100%	100%
	Pyrazinamide	0%	0%
	Imipenem-cilastatin *OR* meropenem, with clavulanic acid	Unknown	
	Amikacin *OR* streptomycin	AMK 2%; SM 0%	AMK 5%; SM 0%
	Ethionamide *OR* prothionamide	0%	0%
	*p-*Aminosalicylic acid	80%**	80%**

An all-oral regimen should comprise all three group A agents and at least one group B agent, such that at least four likely effective drugs are included in the initial phase of treatment. If only one or two group A agents are used, both group B agents should be included in the regimen. Group C agents should be used when an effective regimen (four likely effective agents) cannot be constituted with group A and B drugs. Further information and specifications can be found in [64]

*An additional 5% of strains have emerging fluoroquinolone resistance, which is not reflected by this number

**Based on phenotypic resistance observed in an overlapping cohort [4]

While these data represent a convenience set, we are confident, based on previous [4] and additional (Heupink, manuscript in preparation) work, that this is a representative sampling of the true population structure of AA1SA strains. Although the study lacks direct evidence of treatment effectivity due to the absence of any treatment history or outcome data, most of the frequently occurring mutations described here have been well-described for their roles in drug resistance.

Unfortunately, the data analysed were too limited (genetically similar) to support the findings on a genetically inferred timescale, with insufficient correlation between genetic divergence and sampling time. Our time tree (Additional file 7), generated using published mutation rates [11, 67, 68], suggests that most drug resistance conferring mutations in AA1SA isolates emerged at time points very close to or even before the particular drug’s introduction into routine care. The latter is difficult to explain given the absence of a selective pressure. One explanation is that the mutation rate the AA1SA clade is different to previously published mutation rates [69]. However, parallels can be drawn between the sequence of early drug resistance acquisition and introduction of the different drugs, for example relating to ETH, SM and INH.

Due to the strong influence of drug resistance mutations, we are unable to distinguish between programmatic selection and actual fitness advantage potentially conferred by these mutations regardless of treatment pressure. However, it is clear that drug resistance mutations and possibly additional mutations influence the way the epidemic is shaped.

## Conclusion

We investigated a unique clade of atypical Beijing (AA1SA) isolates from South Africa to address two questions: which factors allow these strains to gain resistance to virtually all available drugs on multiple occasions despite supposed fitness cost associated with drug resistance, and why are some of them so successful in terms of transmission?

In this exploratory work, we identified various genomic mutations that may lie at the root of the problem and warrant further investigation. However, it appears that the driver of this increased resistance acquisition and transmission may be largely programmatic, rather than physiological. Our results suggest that a previously undescribed low-level SM resistance causing *gidB* mutation likely predisposed to high-level SM resistance acquisition, followed by additional resistance acquisition to all first-line drugs. Furthermore, an unexpected deeply rooted *ethA* mutation would not be detected under current South African diagnostic algorithms [70], with the potential to compromise an ETH-containing second-line regimen. In addition, we found that in AA1SA strains, *inhA* promoter mutations do not contribute a drug resistance phenotype, but rather appear to increase the fitness and transmissibility, requiring further investigation.

These results also demonstrate that known exposure to a drug is not an adequate indicator of resistance (e.g. ETH, in AA1SA, or even more currently relevant, BDQ) and it emphasises the risk of amplifying resistance as a result of treating TB without knowledge of the full resistance profile.

The development and spread of beyond-XDR-TB is a phenomenon that is likely to occur repeatedly, as we demonstrate it already has, demanding urgent attention. Despite the promise of new drugs such as BDQ and DLM, these drugs must be used as part of an evidence-based, effective regimen. It is therefore imperative that early reflex diagnostics be rolled out to aid the design of appropriate, tailored treatment strategies. We support the development of WGS technologies to accomplish accurate, comprehensive resistance prediction.

## Supplementary information

**Additional file 1.** Genes observed in this study to carry drug resistance conferring mutations.

**Additional file 2.** Detailed record of sequencing metrics as well as sampling date, origin and genotypic classification for each isolate.

**Additional file 3.** SNP distance matrix. Each cell represents the number of single nucleotide differences between two isolates, as indicated in the column header and leading column.

**Additional file 4.** AA1SA defining variants. Each variant listed occurs in all investigated AA1SA isolates, but not in any other strain investigated in this study.

**Additional file 5.** Tables indicating the nucleotide- and amino acid changes that occur in all members of each of the AA1SA clades A through C, as well as additional information regarding gene function and Provean predictions.

**Additional file 6.** Number of isolates with underlying (read frequency < 0.8) resistance.

**Additional file 7.** S7a Time tree methods and caption to additional file (Time tree).S7b. Figure demonstrating the time tree, with estimated dates of divergence indicated.

**Additional file 8.** Automated sequence analysis pipeline developed by Dr. R.G. van der Merwe.

**Additional file 9.** Python script to compare variants between different isolates or groups of isolates, developed by Dr. R.G. van der Merwe.

**Additional file 10.** Python script to generate a SNP distance matrix, developed by Dr. R.G. van der Merwe.

## Data Availability

Newly sequenced data of the clinical isolates originating from the EC and WC are deposited in the European Nucleotide Archive (ENA; PRJEB35725). Additional sequences derived from other publications are deposited in the ENA under the study accessions PRJEB7281 (https://www.ebi.ac.uk/ena/data/search?query=PRJEB7281) and PRJEB14199 (https://www.ebi.ac.uk/ena/data/view/PRJEB14199) as well as Sequence Read Archive NCBI under the identifiers PRJNA183624 (https://www.ncbi.nlm.nih.gov/bioproject/?term=PRJNA183624) and PRJNA235615 (https://www.ncbi.nlm.nih.gov/bioproject/?term=PRJNA235615). In-house scripts developed by Dr. R.G. van der Merwe were used to automate processing of raw sequencing reads (Additional file 8), compare variants between isolates or groups of isolates (Additional file 9) and generate a SNP distance matrix (Additional file 10).

## References

[1] Migliori GB, De Iaco G, Besozzi G, Centis R, Cirillo DM (2007). First tuberculosis cases in Italy resistant to all tested drugs. Euro Surveill Bull Eur sur les Mal Transm.

[2] Velayati AA, Masjedi MR, Farnia P, Tabarsi P, Ghanavi J, Ziazarifi AH (2009). Emergence of new forms of totally drug-resistant tuberculosis bacilli: super extensively drug-resistant tuberculosis or totally drug-resistant strains in Iran. Chest..

[3] Udwadia ZF, Amale RA, Ajbani KK, Rodrigues C (2012). Totally drug-resistant tuberculosis in India. Clin Infect Dis an Off Publ Infect Dis Soc Am.

[4] Klopper Marisa, Warren Robin Mark, Hayes Cindy, Gey van Pittius Nicolaas Claudius, Streicher Elizabeth Maria, Müller Borna, Sirgel Frederick Adriaan, Chabula-Nxiweni Mamisa, Hoosain Ebrahim, Coetzee Gerrit, David van Helden Paul, Victor Thomas Calldo, Trollip André Phillip (2013). Emergence and Spread of Extensively and Totally Drug-Resistant Tuberculosis, South Africa. Emerging Infectious Diseases.

[5] Dheda K, Gumbo T, Gandhi NR, Murray M, Theron G, Udwadia Z (2014). Global control of tuberculosis: from extensively drug-resistant to untreatable tuberculosis. Lancet Respir Med.

[6] Glynn JR, Whiteley J, Bifani PJ, Kremer K, van Soolingen D (2002). Worldwide occurrence of Beijing/W strains of Mycobacterium tuberculosis: a systematic review. Emerg Infect Dis.

[7] Hanekom M, van der Spuy GD, Streicher E, Ndabambi SL, McEvoy CRE, Kidd M (2007). A recently evolved sublineage of the Mycobacterium tuberculosis Beijing strain family is associated with an increased ability to spread and cause disease. J Clin Microbiol.

[8] Merker M, Barbier M, Cox H, Rasigade J-P, Feuerriegel S, Kohl TA (2018). Compensatory evolution drives multidrug-resistant tuberculosis in Central Asia. Elife..

[9] Lasunskaia E, Ribeiro SCM, Manicheva O, Gomes LL, Suffys PN, Mokrousov I (2010). Emerging multidrug resistant Mycobacterium tuberculosis strains of the Beijing genotype circulating in Russia express a pattern of biological properties associated with enhanced virulence. Microbes Infect.

[10] Marais B. J., Mlambo C. K., Rastogi N., Zozio T., Duse A. G., Victor T. C., Marais E., Warren R. M. (2013). Epidemic Spread of Multidrug-Resistant Tuberculosis in Johannesburg, South Africa. Journal of Clinical Microbiology.

[11] Merker M, Blin C, Mona S, Duforet-Frebourg N, Lecher S, Willery E (2015). Evolutionary history and global spread of the Mycobacterium tuberculosis Beijing lineage. Nat Genet.

[12] Filliol I, Motiwala AS, Cavatore M, Qi W, Hazbón MH, Bobadilla del Valle M (2006). Global phylogeny of Mycobacterium tuberculosis based on single nucleotide polymorphism (SNP) analysis: insights into tuberculosis evolution, phylogenetic accuracy of other DNA fingerprinting systems, and recommendations for a minimal standard SNP set. J Bacteriol.

[13] Coll F, McNerney R, Guerra-Assunção JA, Glynn JR, Perdigão J, Viveiros M (2014). A robust SNP barcode for typing Mycobacterium tuberculosis complex strains. Nat Commun.

[14] Shitikov E, Kolchenko S, Mokrousov I, Bespyatykh J, Ischenko D, Ilina E (2017). Evolutionary pathway analysis and unified classification of East Asian lineage of Mycobacterium tuberculosis. Sci Rep.

[15] Plikaytis BB, Marden JL, Crawford JT, Woodley CL, Butler WR, Shinnick TM (1994). Multiplex PCR assay specific for the multidrug-resistant strain W of Mycobacterium tuberculosis. J Clin Microbiol.

[16] Iwamoto T, Yoshida S, Suzuki K, Wada T (2008). Population structure analysis of the Mycobacterium tuberculosis Beijing family indicates an association between certain sublineages and multidrug resistance. Antimicrob Agents Chemother.

[17] Ribeiro SCM, Gomes LL, Amaral EP, Andrade MRM, Almeida FM, Rezende AL (2014). Mycobacterium tuberculosis strains of the modern sublineage of the Beijing family are more likely to display increased virulence than strains of the ancient sublineage. J Clin Microbiol.

[18] Dou H-Y, Tseng F-C, Lu J-J, Jou R, Tsai S-F, Chang J-R (2008). Associations of Mycobacterium tuberculosis genotypes with different ethnic and migratory populations in Taiwan. Infect Genet Evol J Mol Epidemiol Evol Genet Infect Dis.

[19] Maeda S, Hang NTL, Lien LT, Thuong PH, Hung NV, Hoang NP (2014). Mycobacterium tuberculosis strains spreading in Hanoi, Vietnam: Beijing sublineages, genotypes, drug susceptibility patterns, and host factors. Tuberculosis..

[20] Millet J, Miyagi-Shiohira C, Yamane N (2012). High-resolution MIRU-VNTRs typing reveals the unique nature of Mycobacterium tuberculosis Beijing genotype in Okinawa. Japan Infect Genet Evol.

[21] Chihota VN, Muller B, Mlambo CK, Pillay M, Tait M, Streicher EM (2012). Population structure of multi- and extensively drug-resistant Mycobacterium tuberculosis strains in South Africa. J Clin Microbiol.

[22] Andersson DI, Levin BR (1999). The biological cost of antibiotic resistance. Curr Opin Microbiol.

[23] Gagneux S, Long CD, Small PM, Van T, Schoolnik GK, Bohannan BJM (2006). The competitive cost of antibiotic resistance in *Mycobacterium tuberculosis*. Science (80- ).

[24] Dheda Keertan, Limberis Jason D, Pietersen Elize, Phelan Jody, Esmail Aliasgar, Lesosky Maia, Fennelly Kevin P, te Riele Julian, Mastrapa Barbara, Streicher Elizabeth M, Dolby Tania, Abdallah Abdallah M, Ben-Rached Fathia, Simpson John, Smith Liezel, Gumbo Tawanda, van Helden Paul, Sirgel Frederick A, McNerney Ruth, Theron Grant, Pain Arnab, Clark Taane G, Warren Robin M (2017). Outcomes, infectiousness, and transmission dynamics of patients with extensively drug-resistant tuberculosis and home-discharged patients with programmatically incurable tuberculosis: a prospective cohort study. The Lancet Respiratory Medicine.

[25] Cohen Keira A., Abeel Thomas, Manson McGuire Abigail, Desjardins Christopher A., Munsamy Vanisha, Shea Terrance P., Walker Bruce J., Bantubani Nonkqubela, Almeida Deepak V., Alvarado Lucia, Chapman Sinéad B., Mvelase Nomonde R., Duffy Eamon Y., Fitzgerald Michael G., Govender Pamla, Gujja Sharvari, Hamilton Susanna, Howarth Clinton, Larimer Jeffrey D., Maharaj Kashmeel, Pearson Matthew D., Priest Margaret E., Zeng Qiandong, Padayatchi Nesri, Grosset Jacques, Young Sarah K., Wortman Jennifer, Mlisana Koleka P., O'Donnell Max R., Birren Bruce W., Bishai William R., Pym Alexander S., Earl Ashlee M. (2015). Evolution of Extensively Drug-Resistant Tuberculosis over Four Decades: Whole Genome Sequencing and Dating Analysis of Mycobacterium tuberculosis Isolates from KwaZulu-Natal. PLOS Medicine.

[26] Coll F, Mallard K, Preston MD, Bentley S, Parkhill J, McNerney R (2012). SpolPred: rapid and accurate prediction of Mycobacterium tuberculosis spoligotypes from short genomic sequences. Bioinformatics..

[27] Warren R., de Kock M., Engelke E., Myburgh R., Gey van Pittius N., Victor T., van Helden P. (2006). Safe Mycobacterium tuberculosis DNA Extraction Method That Does Not Compromise Integrity. Journal of Clinical Microbiology.

[28] Black P, de Vos M, Louw G, van der Merwe R, Dippenaar A, Streicher E (2015). Whole genome sequencing reveals genomic heterogeneity and antibiotic purification in Mycobacterium tuberculosis isolates. BMC Genomics.

[29] Bolger AM, Lohse M, Usadel B (2014). Trimmomatic: a flexible trimmer for Illumina sequence data. Bioinformatics..

[30] Li H, Durbin R (2009). Fast and accurate short read alignment with Burrows-Wheeler transform. Bioinformatics..

[31] Novocraft.com. Novoalign.

[32] SMALT - Wellcome Trust Sanger Institute [Internet]. [cited 2015 Aug 6]. Available from: https://www.sanger.ac.uk/resources/software/smalt/

[33] McKenna A, Hanna M, Banks E, Sivachenko A, Cibulskis K, Kernytsky A (2010). The Genome Analysis Toolkit: a MapReduce framework for analyzing next-generation DNA sequencing data. Genome Res.

[34] Coll F, McNerney R, Preston MD, Guerra-Assunção JA, Warry A, Hill-Cawthorne G, et al. Rapid determination of anti-tuberculosis drug resistance from whole-genome sequences. Genome Med. 2015;7:51. 10.1186/s13073-015-0164-0. 10.1186/s13073-015-0164-0 PMC4446134 26019726

[35] Carver T, Berriman M, Tivey A, Patel C, Böhme U, Barrell BG (2008). Artemis and ACT: viewing, annotating and comparing sequences stored in a relational database. Bioinformatics..

[36] Abeel T, Van Parys T, Saeys Y, Galagan J, Van de Peer Y (2012). GenomeView: a next-generation genome browser. Nucleic Acids Res.

[37] Coll F, McNerney R, Preston MD, Guerra-Assunção JA, Warry A, Hill-Cawthorne G (2015). Rapid determination of anti-tuberculosis drug resistance from whole-genome sequences. Genome Med.

[38] Nguyen L-T, Schmidt HA, von Haeseler A, Minh BQ (2015). IQ-TREE: a fast and effective stochastic algorithm for estimating maximum-likelihood phylogenies. Mol Biol Evol.

[39] Kalyaanamoorthy S, Minh BQ, Wong TKF, von Haeseler A, Jermiin LS (2017). ModelFinder: fast model selection for accurate phylogenetic estimates. Nat Methods.

[40] Coscolla M, Lewin A, Metzger S, Maetz-Rennsing K, Calvignac-Spencer S, Nitsche A (2013). Novel Mycobacterium tuberculosis complex isolate from a wild chimpanzee. Emerg Infect Dis.

[41] Yu G, Smith DK, Zhu H, Guan Y, Lam TT-Y. ggtree: an r package for visualization and annotation of phylogenetic trees with their covariates and other associated data. McInerny G, editor. Methods Ecol Evol 2017;8(1):28–36.

[42] Choi Y, Sims GE, Murphy S, Miller JR, Chan AP. Predicting the functional effect of amino acid substitutions and indels. de Brevern AG, editor. PLoS One. 2012;7(10):e46688. 10.1371/journal.pone.0046688 PMC3466303 23056405

[43] Lew JM, Kapopoulou A, Jones LM, Cole ST (2011). TubercuList--10 years after. Tuberculosis (Edinb).

[44] Tsolaki A. G., Gagneux S., Pym A. S., Goguet de la Salmoniere Y.-O. L., Kreiswirth B. N., Van Soolingen D., Small P. M. (2005). Genomic Deletions Classify the Beijing/W Strains as a Distinct Genetic Lineage of Mycobacterium tuberculosis. Journal of Clinical Microbiology.

[45] Tsolaki AG, Hirsh AE, DeRiemer K, Enciso JA, Wong MZ, Hannan M (2004). Functional and evolutionary genomics of Mycobacterium tuberculosis: insights from genomic deletions in 100 strains. Proc Natl Acad Sci U S A.

[46] Coll F, Phelan J, Hill-Cawthorne GA, Nair MB, Mallard K, Ali S (2018). Genome-wide analysis of multi- and extensively drug-resistant Mycobacterium tuberculosis. Nat Genet.

[47] Springer B, Lucke K, Calligaris-Maibach R, Ritter C, Bottger EC (2009). Quantitative drug susceptibility testing of Mycobacterium tuberculosis by use of MGIT 960 and EpiCenter instrumentation. J Clin Microbiol.

[48] WHO | Technical report on critical concentrations for TB drug susceptibility testing of medicines used in the treatment of drug-resistant TB. WHO. 2018;

[49] Li X-Z, Zhang L, Nikaido H (2004). Efflux pump-mediated intrinsic drug resistance in Mycobacterium smegmatis. Antimicrob Agents Chemother.

[50] Plater R, Robinson JA (1992). Cloning and sequence of a gene encoding macrotetrolide antibiotic resistance from Streptomyces griseus. Gene..

[51] Zahrt TC, Wozniak C, Jones D, Trevett A (2003). Functional analysis of the Mycobacterium tuberculosis MprAB two-component signal transduction system. Infect Immun.

[52] Senaratne RH, Mobasheri H, Papavinasasundaram KG, Jenner P, Lea EJ, Draper P (1998). Expression of a gene for a porin-like protein of the OmpA family from Mycobacterium tuberculosis H37Rv. J Bacteriol.

[53] de Vos M, Müller B, Borrell S, Black PA, van Helden PD, Warren RM (2013). Putative compensatory mutations in the rpoC gene of rifampin-resistant Mycobacterium tuberculosis are associated with ongoing transmission. Antimicrob Agents Chemother.

[54] Wong SY, Lee JS, Kwak HK, Via LE, Boshoff HIM, Barry CE (2011). Mutations in gidB confer low-level streptomycin resistance in Mycobacterium tuberculosis. Antimicrob Agents Chemother.

[55] Porteous JB (1959). The treatment of pulmonary tuberculosis. S Afr Med J.

[56] Müller B, Streicher EM, Hoek KGP, Tait M, Trollip A, Bosman ME (2011). inhA promoter mutations: a gateway to extensively drug-resistant tuberculosis in South Africa?. Int J Tuberc lung Dis Off J Int Union against Tuberc Lung Dis.

[57] DeBarber AE, Mdluli K, Bosman M, Bekker LG, Barry CE (2000). Ethionamide activation and sensitivity in multidrug-resistant Mycobacterium tuberculosis. Proc Natl Acad Sci U S A.

[58] Walker TM, Merker M, Knoblauch AM, Helbling P, Schoch OD, van der Werf MJ (2018). A cluster of multidrug-resistant Mycobacterium tuberculosis among patients arriving in Europe from the Horn of Africa: a molecular epidemiological study. Lancet Infect Dis.

[59] Salinger PL, Dormer BA (1972). Rifampicin, ethambutol, ethionamide and hydronsan in advanced pulmonary tuberculosis. S Afr Med J.

[60] B Marshall Clarke BG, Dph C, Consultant Chest Physician D, Hospital K. Chronic pulmonary tuberculosis treatment with ethionamide combined with cycloserine or oxytetracycline. 10.1136/bmj.1.5226.636 PMC1953486 13693888

[61] South African Department of Health (2014). National tuberculosis management guidelines.

[62] Andries K, Villellas C, Coeck N, Thys K, Gevers T, Vranckx L, et al. Acquired resistance of *Mycobacterium tuberculosis* to bedaquiline. van Veen HW, editor. PLoS One. 2014;9(7):e102135. 10.1371/journal.pone.0102135 PMC4092087 25010492

[63] Villellas C, Coeck N, Meehan CJ, Lounis N, de Jong B, Rigouts L (2017). Unexpected high prevalence of resistance-associated Rv0678 variants in MDR-TB patients without documented prior use of clofazimine or bedaquiline. J Antimicrob Chemother.

[64] WHO. Rapid communication: key changes to treatment of multidrug- and rifampicin-resistant tuberculosis (MDR/RR-TB). 2018.

[65] von Groote-Bidlingmaier Florian, Patientia Ramonde, Sanchez Epifanio, Balanag Vincent, Ticona Eduardo, Segura Patricia, Cadena Elizabeth, Yu Charles, Cirule Andra, Lizarbe Victor, Davidaviciene Edita, Domente Liliana, Variava Ebrahim, Caoili Janice, Danilovits Manfrid, Bielskiene Virgaine, Staples Suzanne, Hittel Norbert, Petersen Carolyn, Wells Charles, Hafkin Jeffrey, Geiter Lawrence J, Gupta Rajesh (2019). Efficacy and safety of delamanid in combination with an optimised background regimen for treatment of multidrug-resistant tuberculosis: a multicentre, randomised, double-blind, placebo-controlled, parallel group phase 3 trial. The Lancet Respiratory Medicine.

[66] Polsfuss S, Hofmann-Thiel S, Merker M, Krieger D, Niemann S, Rüssmann H, et al. Emergence of low-level delamanid and bedaquiline resistance during extremely drug-resistant tuberculosis treatment. Clin Infect Dis. 2019; 10.1093/cid/ciz074 30933266

[67] Sagulenko P, Puller V, Neher RA. TreeTime: maximum-likelihood phylodynamic analysis. Virus Evol 2018 1;4(1). 10.1093/ve/vex042 PMC5758920 29340210

[68] Roetzer A, Diel R, Kohl TA, Rückert C, Nübel U, Blom J, et al. Whole genome sequencing versus traditional genotyping for investigation of a *Mycobacterium tuberculosis* outbreak: a longitudinal molecular epidemiological study. Neyrolles O, editor. PLoS Med. 2013;10(2):e1001387. 10.1371/journal.pmed.1001387 PMC3570532 23424287

[69] Menardo F, Duchêne S, Brites D, Gagneux S. The molecular clock of *Mycobacterium tuberculosis*. Biek R, editor. PLOS Pathog. 2019;15(9):e1008067. 10.1371/journal.ppat.1008067 PMC6759198 31513651

[70] Interim clinical guidance for the implementation of injectable-free regimens for rifampicin-resistant tuberculosis in adults, adolescents and children 2 | P a g e.

