# Supplementary material for: A landscape of genomic alterations at the root of a near-untreatable tuberculosis epidemic
Source: BMC Med. 2020 Feb 21;18:24. doi: 10.1186/s12916-019-1487-2 (PMC6998097; doi:10.1186/s12916-019-1487-2)
Supplement: Supplementary file 5 — Additional file 5. Tables indicating the nucleotide- and amino acid changes that occur in all members of each of the AA1SA clades A through C, as well as additional information regarding gene function and Provean predictions. [file 12916_2019_1487_MOESM5_ESM.docx]

Table 3.1: Variants defining clade A, B and C collectively, as per figure 1.

| **Genomic position** | **Base change** | **Amino acid change** | **Gene** | **Product** | **Function** | **Functional category** | **PROVEAN analysis** | **Essentiality  (yes/no)** |
| --- | --- | --- | --- | --- | --- | --- | --- | --- |
| 1124640 | G > A | V309V | *Rv1006* | Unknown protein | Unknown | conserved hypotheticals | synonymous |  |
| 1269567 | A > G | L131S | *echA10 (Rv1142c)* | Probable enoyl-CoA hydratase | Oxidize fatty acids | lipid metabolism | -4.271 (Deleterious) | No |
| 2155168 | C > G | S315T | *katG (Rv1908c)* | Catalase-peroxidase-peroxynitritase T | Catalase, peroxidase, peroxynitritase | virulence, detoxification, adaptation | -2.853 (Deleterious) | No |
| 3457119 | C > T | S453S | *fadD13 (Rv3089)* | Fatty-acyl-CoA synthetase | Involved in lipid degradation | lipid metabolism | synonymous | No |
| 3967599 | A > G | L74L | *Rv3530c* | Possible oxidoreductase | Unknown | intermediary metabolism and respiration | synonymous | No |
| 4051189 | C > A | A565A | *ftsH (Rv3610c)* | Membrane-bound protease | Regulatory role in stress response | cell wall and cell processes | synonymous | Yes |
| 4127994 | G > A | A244A | *cyp137 (Rv3685c)* | Probable cytochrome P450 | Heme-thiolate monooxygenases | intermediary metabolism and respiration | synonymous | No |
| 4407967 | A > G | L79S | *gidB (Rv3919c)* | Probable glucose-inhibited division protein | Unknown | cell wall and cell processes | -5.297 (Deleterious) | No |
| 1890038 | del G | frameshift @ codon 671 | *pks9 (Rv1664)* | Probable polyketide synthase | Intermediate steps for polyketide synthesis | lipid metabolism | frameshift | No |

Table 3.2 Variants defining clades A1 and A2

| **Genomic position** | **Base change** | **Amino acid change** | **Gene** | **Product** | **Function** | **Functional category** | **PROVEAN analysis** | **Occurrence** |
| --- | --- | --- | --- | --- | --- | --- | --- | --- |
| 880387 | C > T | H350Y | *Rv0785* | Conserved protein | Unknown | conserved hypotheticals | 0.073 (Neutral) | Clade A1, A2 |
| 2289202 | T > C | C14R | *pncA (Rv2043c)* | Pyrazinamidase/  nicotinamidase | Converts amides to corresponding acid | intermediary metabolism and respiration | -11.520 (Deleterious) | Clade A1, A2 |
| 2605150 | G > A | A15T | *mez (Rv2332)* | Probable [NAD] dependent malate oxidoreductase | Oxidative decarboxylation of malate into pyruvate | intermediary metabolism and respiration | 0.829 (Neutral) | Clade A1, A2 |
| 3511368 | G > T | intergenic | *PPE52 (-)* \|*nuoA (+)* | - | - | - | intergenic | Clade A1, A2 |
| 4247431 | G > C | M306I | *embB (Rv3795)* | arabinosyltransferase | Cell wall arabinan biosynthesis | cell wall and cell processes | -3.181 (Deleterious) | Clade A1, A2; homoplastic |
| 761155 | C > T | S450L | *rpoB (Rv0667)* | DNA-directed RNA polymerase | Catalyzes the transcription of DNA into RNA | information pathways | -5.896 (Deleterious) | Clade A1, A2; homoplastic |
| 764817 | T > G | V483G | *rpoC (Rv0668)* | DNA-directed RNA polymerase | Catalyzes the transcription of DNA into RNA | information pathways | -6.997 (Deleterious) | Clade A1, A2; homoplastic |
| 410962 | A > G | H42R | *iniA (Rv0342)* | Isoniazid inducible gene protein | Unknown | cell wall and cell processes | -0.142 (Neutral) | Clade A1 |
| 2722670 | G > A | E213E | *Rv2425c* | Conserved hypothetical protein | Unknown | conserved hypotheticals | synonymous | Clade A1 |
| 4269089 | G > A | A249T | *ubiA (Rv3806c)* | Decaprenylphosphoryl-5-phosphoribose (DPPR) synthase | Involved in arabinogalactan synthesis | cell wall and cell processes | -0.689 (Neutral) | Clade A1 |
| 1673425 | C > T | position -15 | *Rv1482c (-)* \| *mabA (+)* | (*inhA* promoter) | Upregulates fabG1, inhA, hemZ | - | intergenic | Clade A1; homoplastic |

Table 3.3 Variants defining clade B

| **Genomic position** | **Base change** | **Amino acid change** | **Gene** | **Product** | **Function** | **Functional category** | **PROVEAN analysis** |
| --- | --- | --- | --- | --- | --- | --- | --- |
| 737015 | G > A | D63D | *mmaA4 (Rv0642c)* | Methoxy mycolic acid synthase | Mycolic acids modification | lipid metabolism | synonymous |
| 761110 | A > T | D435V | *rpoB (Rv0667)* | DNA-directed RNA polymerase | Catalyzes the transcription of DNA into RNA | information pathways | -8.984 (Deleterious) |
| 1471474 | G > A | G385D | *murA (Rv1315)* | Probable UDP-N-acetylglucosamine 1-carboxyvinyl-transferase | Peptidoglycan biosynthesis | cell wall and cell processes | -5.898 (Deleterious) |
| 1673423 | G > T | position -17 | *Rv1482c (-)* \| *mabA (+)* | (*inhA* promoter) | Upregulates fabG1, inhA, hemZ | - | intergenic |
| 2266314 | T > C | I109T | *Rv2019* | Conserved protein | Unknown | conserved hypothetical | -1.941 (Neutral) |
| 3204705 | G > A | G176G | *viuB (Rv2895c)* | Possible mycobactin utilization protein | Intracellular removal of iron from iron-mycobactin complex | Intermed. metabolism and respiration | synonymous |
| 4247431 | G > A | M306I | *embB (Rv3795)* | Arabinosyltrans-ferase | Cell wall arabinan biosynthesis | cell wall and cell processes | -3.181 (Deleterious) |
| 2288724 | ins C | Frame-shift @ codon 173 | *pncA (Rv2043c)* | Pyrazinamidase/ nicotinamidase | Convert amide to correspond-ding acid | Intermed. metabolism and respiration | frameshift |
| 3136977 | ins T | Frame-shift @ codon 12 | *vapC22 (Rv2829c)* | Toxin | Unknown | virulence, detox., adaptation | frameshift |

Table 3.4 Variants defining clade C

| **Genomic position** | **Base change** | **Amino acid change** | **Gene** | | **Product** | **Function** | | **Functional category** | |  | **PROVEAN analysis** | | |
| --- | --- | --- | --- | --- | --- | --- | --- | --- | --- | --- | --- | --- | --- |
| 145365 | C > T | G439G | | fadD7 (Rv0119) | Fatty-acid-CoA ligase | Involved in lipid degradation. | lipid metabolism | | - | | |  |  |
| 627739 | C > T | V164V | | galE3 (Rv0536) | UDP-glucose 4-epimerase | Galactose metabolism | intermediary metabolism and respiration | | - | | |  |  |
| 1240760 | C > T | P192S | | Rv1115 | Possible exported protein | Unknown | cell wall and cell processes | | -0.912 (Neutral) | | |  |  |
| 1674685 | A > T | T162S | | inhA (Rv1484) | NADH-dependent enoyl-ACP reductase | Mycolic acid biosynthesis | lipid metabolism | | 1.838 (Neutral) | | |  |  |
| 2111616 | Del T | Frameshift @ codon 164 | | Rv1864c | Conserved protein | Function unknown | conserved hypotheticals | | frameshift | | |  |  |
