# Supplementary material for: A landscape of genomic alterations at the root of a near-untreatable tuberculosis epidemic
Source: BMC Med. 2020 Feb 21;18:24. doi: 10.1186/s12916-019-1487-2 (PMC6998097; doi:10.1186/s12916-019-1487-2)
Supplement: Supplementary file 7 — Additional file 7. S7a Time tree methods and caption to additional file (Time tree).S7b. Figure demonstrating the time tree, with estimated dates of divergence indicated. [file 12916_2019_1487_MOESM7_ESM.zip › Additional file S7a Time tree methods and captionR3.docx]

We attempted a time calibrated phylogeny, but due to insufficient temporal signal to estimate a mutation rate from the data, we instead used a previously published mutation rate for the time calibration. TimeTree v0.5.3 was used to time-calibrate the phylogeny using a molecular clock of 1 x 10^-7^ substitutions per nucleotide site per year (95% confidence interval: 0.6 x 10^-7^ – 1.5 x 10^-7^ (1–3), and sample dates were provided where available.

1. Sagulenko P, Puller V, Neher RA. TreeTime: Maximum-likelihood phylodynamic analysis. Virus Evol. 2018 Jan 1;4(1).

2. Roetzer A, Diel R, Kohl TA, Rückert C, Nübel U, Blom J, et al. Whole Genome Sequencing versus Traditional Genotyping for Investigation of a Mycobacterium tuberculosis Outbreak: A Longitudinal Molecular Epidemiological Study. Neyrolles O, editor. PLoS Med. 2013 Feb 12;10(2):e1001387.

3. Merker M, Blin C, Mona S, Duforet-Frebourg N, Lecher S, Willery E, et al. Evolutionary history and global spread of the Mycobacterium tuberculosis Beijing lineage. Nat Genet. 2015 Mar;47(3):242–9.

Additional file S7 Figure caption:

Time-calibrated phylogeny of Beijing genotype isolates (based on Figure 1 in the main text), using a molecular clock of 1 x 10^-7^ substitutions per nucleotide site per year (95% confidence interval: 0.6 x 10^-7^ – 1.5 x 10^-7^), showing branching points corresponding to drug-resistance acquisition prior to introduction of the drugs in many instances. For more information please contact the author.
