# Supplementary figures and images for: A landscape of genomic alterations at the root of a near-untreatable tuberculosis epidemic
Source: BMC Med. 2020 Feb 21;18:24. doi: 10.1186/s12916-019-1487-2 (PMC6998097; doi:10.1186/s12916-019-1487-2)

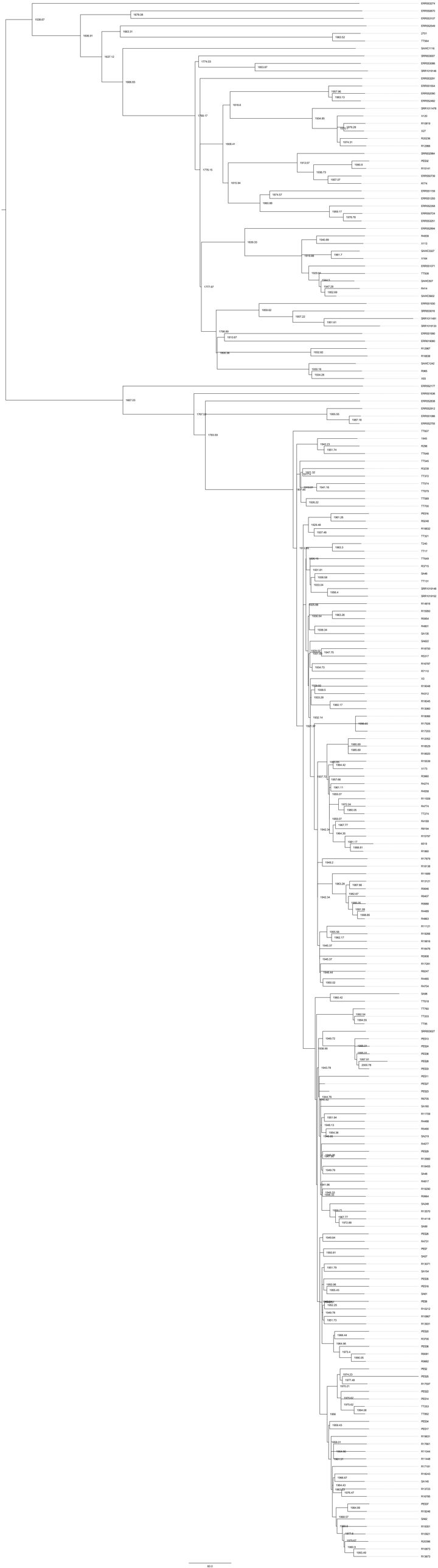

Supplement: Supplementary file 7 — Additional file 7. S7a Time tree methods and caption to additional file (Time tree).S7b. Figure demonstrating the time tree, with estimated dates of divergence indicated. [file 12916_2019_1487_MOESM7_ESM.zip › Additional file S7b timetree.NodeDateR3.pdf]
